# Cancer care patterns in South Korea: Types of hospital where patients receive care and outcomes using national health insurance claims data

**DOI:** 10.1002/cam4.6093

**Published:** 2023-05-18

**Authors:** Dong‐Woo Choi, Sun Jung Kim, Seungju Kim, Dong Wook Kim, Wonjeong Jeong, Kyu‐Tae Han

**Affiliations:** ^1^ Cancer Big Data Center, National Cancer Control Institute, National Cancer Center Goyang Republic of Korea; ^2^ Department of Health Administration and Management, College of Medical Science Soonchunhyang University Asan‐Si Republic of Korea; ^3^ Department of Nursing, College of Nursing The Catholic University of Korea Seoul Republic of Korea; ^4^ Department of Information and Statistics RINS, Gyeongsang National University Jinju‐si Republic of Korea; ^5^ Cancer Knowledge & Information Center, National Cancer Control Institute, National Cancer Center Goyang Republic of Korea; ^6^ Division of Cancer Control & Policy National Cancer Control Institute, National Cancer Center Goyang Republic of Korea

**Keywords:** cancer care, cancer outcomes, health service research, healthcare delivery system, trajectory modeling

## Abstract

**Background:**

Although strengthening coverage has improved cancer care, there are concerns related to medical distortion. Previous studies have only examined whether patients visit a specific hospital, and not the continuum of patients with cancer, resulting in a lack of evidence in South Korea. This study aimed to investigate the patterns in hospital type for cancer care and analyze their association with outcomes.

**Methods:**

The data for this study were obtained from the National Health Insurance Services Sampled Cohort database. This study included patients with four types of cancer (top four cancer incidence in 2020): gastric (3353), colorectal (2915), lung (1351), and thyroid (5158) cancer. The latent class mixed model was used to investigate cancer care patterns, and multiple regression or survival analysis was performed to examine medical cost, length of stay (LOS), and mortality.

**Results:**

The patterns in each cancer type were classified into two to four classes, namely, mainly visited clinics or hospitals, mainly visited general hospitals, mainly visited tertiary hospitals (MT), and tertiary to general hospitals through trajectory modeling based on the utilization of cancer care. Compared to the MT pattern, other patterns were generally associated with higher cost, LOS, and mortality.

**Conclusion:**

The patterns found in this study may be a more realistic way of defining patients with cancer in South Korea compared to previous studies, and its association‐related outcomes may be used as a basis to address problems in the healthcare system and prepare alternatives for patients with cancer. Future studies should review cancer care patterns related to other factors such as regional distribution.

## 1 BACKGROUND

Cancer has been considered the most common serious disease affecting physical, psychological, and socioeconomic health in South Korea over the past few decades, and many health policies have been introduced to control its effects. The most representative policy is a co‐payment reduction program under the National Health Insurance (NHI) for cancer and other severe diseases. From September 2005 to December 2009, the South Korean government gradually reduced the burden on patients requiring cancer treatment, and the percentage of out‐of‐pocket expenditure decreased from 20%–30% to 5%.^1^ Based on previous studies, related policies and programs had several positive effects on patients with cancer.^2^, ^3^, ^4^ A remarkable improvement has been observed in the survival of patients with cancer (5‐year relative survival rate: 54.1% in 2001–2005 to 71.5% in 2016–2020).^5^ The expansion of insurance coverage for cancer care has significantly assisted economic accessibility, contributed to the reduction of catastrophic medical expenses for patients with cancer,^3^, ^4^ and provided them with various options, resulting in a major change in cancer care along with geographical characteristics and the development of transportation.^6^, ^7^ In addition, NHI covers a significant number of cancer treatments based on clinical and cost effectiveness, and the expansion of health insurance coverage is associated with a significant improvement in cancer care outcomes.^8^

According to South Korea's cancer‐related NHI statistics, healthcare expenditures have significantly increased. Medical cost due to cancer has increased from $1.2 billion in 2004 to approximately $9.2 billion in 2021 (won‐dollar exchange rate in 2021: 1188.8 Korea Won [KRW] = $1), and the proportion of total medical cost has increased significantly (2004: 9.5% to 2021: 11.9% of total medical cost in South Korea).^9^ Strengthening cancer care coverage via the co‐payment reduction program introduced in stages from 2005 to 2009 is associated with patient behaviors. This is because economic accessibility can be improved by reducing patients' out‐of‐pocket burden. With the expectation of receiving high‐quality cancer treatment, patients desire to visit high‐volume or capital area hospitals from the perspective of medical use, resulting in an impact on overall cancer care such as excessive end‐of‐life medical expenditure.^10^, ^11^, ^12^, ^13^ According to the 2021 Health Insurance Review and Assessment statistics, more than half of cancer treatment medical costs occur in capital areas (62.4%). Additionally, 68.2% of cancer‐related medical expenses are spent at tertiary hospitals.^14^

Concerns about the distortion of cancer care such as imbalance of care and its impact on the healthcare market have been raised by professionals and decision makers; however, clear evidence is not available in South Korea.^15^, ^16^, ^17^ Previous research has only examined whether patients visit a specific hospital more than once in view of size and type, disregarding the overall continuum of each patient with cancer.^18^ Additionally, other studies examining the accessibility, such as time‐to‐treatment in cancer care, and efforts to evaluate cancer treatment processes or patterns, such as cancer diagnosis and comparison between hospitals providing cancer treatment, are insufficient.^19^, ^20^, ^21^, ^22^ Therefore, very few studies have examined aspects of cancer care from the perspective of the patient and the effect of cancer care patterns on health outcomes in South Korea.

The present study aimed to investigate the patterns in hospital types for receiving cancer care after the first diagnosis of cancer using NHI claims data and analyze the association between the patterns based on cancer types and outcomes. Based on the findings of this study, alternative policies related to cancer care in South Korea can be formulated to solve the inefficiency of the healthcare delivery system.

## 2 METHODS

### 2.1 Study population

The data used in this study were obtained from the National Health Insurance Services Sampled Cohort database and consisted of 2.2% (*N* = 1,000,000) of individuals who were randomly sampled after stratification according to sex, age, region, type of insurance, and insurance premium among the South Korean population (*N* = 48,222,537 in 2006). They included information on patient characteristics such as demographic and socioeconomic factors, healthcare utilization and treatment details, medical check‐ups, and medical institution characteristics, and sampled individuals' follow‐ups from 2002 to 2015.

**FIGURE 1:**
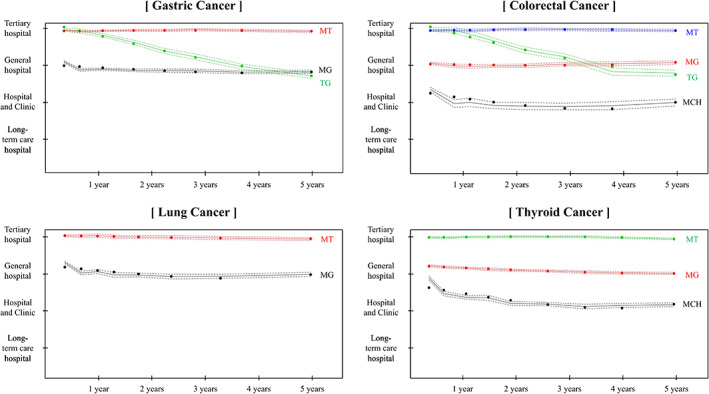
Patterns of cancer care using trajectory modeling according to the type of cancer. † The results of the latent class mixed model investigating the hospitals' patterns with high medical expenses every 3 months considering the model fit using Bayesian information criterion and Akaike information criterion. Only models with at least 5% of the sample were included. MCH: mainly visiting clinic or hospital, MG: mainly visiting general hospital, MT: mainly visiting tertiary hospital, TG: tertiary to general hospital.

This study included patients with four types of cancer (gastric, lung, colorectal, and thyroid cancer; top four cancers with high incidence in 2020, age‐standardized cancer incidence per 100,000, thyroid cancer = 56.8, lung cancer = 56.4, colon cancer = 54.3, and stomach cancer = 51.9).^23^ The types of cancer were defined based on major diagnosis code combinations (International Classification of Diseases‐10: C16 [gastric], C18–C20 [colorectal], C33–C34 [lung], and C73 [thyroid]; *N* = 9128 [gastric], 8278 [colorectal], 6390 [lung], and 8578 [thyroid]).^24^, ^25^ To analyze the cancer care patterns and their effect on patient outcomes, patients diagnosed with other cancer types within 5 years prior to a specific cancer diagnosis were excluded, and only those diagnosed with cancer since 2007 were included to ensure an observation period of more than 5 years as the data started from 2002 and define a new cancer diagnosis. The date of cancer diagnosis was defined as the index date. Patients with cancer who did not have medical records such as hospital characteristics were excluded. In addition, patients with no history of cancer treatment—such as surgery, chemotherapy, and radiation therapy—within 1 year after cancer diagnosis or patients who died within 1 year were excluded to minimize reverse causation. To compare results, including cost, length of stay (LOS), and mortality, the observation period was set to a maximum of 5 years. Even if the patient survived for more than 5 years after cancer, only results within 5 years were measured. Finally, patients with gastric (3353), colorectal (2915), lung (1351), and thyroid (5158) cancer were included in this study to examine cancer care patterns and identify their impact on outcomes (Figure 1).

### 2.2 Variables

#### 2.2.1 Patterns of cancer care

The major variable of interest in this study was cancer care patterns. Cancer care patterns were defined by the type of hospital each patient with cancer mainly visited. First, only those medical records with cancer‐specific insurance claim code (V193) were selected. Second, the hospital with the highest medical expenses was identified by organizing the cancer care medical expenses at each hospital every 3 months. Finally, the latent class mixed model (LCMM), which describes the changes of longitudinal outcomes over time, was used to investigate the patterns of hospitals with high medical expenses every 3 months.^26^ These models were separately adopted based on cancer type, considering the fit of the model using Bayesian Information Criterion and Akaike Information Criterion, and the model was only included if it consisted of at least 5% of the sample to prevent overfitting.^27^ Based on these criteria, model fitting and distribution were tested for one to five patterns for each type of cancer. Consequently, the medical utilization patterns in each type of cancer were classified into two to four classes: mainly visited clinics or hospitals (MCH), mainly visited general hospitals (MH), mainly visited tertiary hospitals (MT), and tertiary to general hospital (TG) (Figure 1; Table S1).

#### 2.2.2 Outcomes of cancer care

The outcome variables in this study were (1) LOS, (2) medical cost (won‐dollar exchange rate was applied as 1188.8 KRW = $1), and (3) 5‐year mortality. LOS and medical cost were defined based on the sum of medical details with a cancer‐specific claim code (V193) after cancer diagnosis, which was converted to the value of 1 year after dividing observation period (up to 1825 days, 5 years) to compare the patterns of patients with cancer.^28^, ^29^, ^30^

1. LOS (days) = (sum of LOS in the medical details with cancer care after cancer diagnosis [days] ÷ sum of observation period [days, up to 1825 days]) × 365
2. Medical cost ($) = (sum of medical cost in the medical details with cancer care after cancer diagnosis [$] ÷ sum of observation period [days, up to 1825 days]) × 365

Five‐year mortality was defined by observing the patients after identifying their first date of diagnosis. Regardless of the cause of death, patients who died within 1825 days after cancer diagnosis were defined as “Deceased” for 5‐year mortality, and the others were defined as “Survived.”

3 Five‐year mortality = whether patients died within 1825 days after cancer diagnosis

#### 2.2.3 Covariates

The covariates included hospital characteristics (type and location of the hospital that provided the first course of treatment), sex, age, insurance coverage types, economic status, residence area, Charlson Comorbidity Index (CCI), year of diagnosis, and cancer treatment types. The characteristics of the hospital which provided the first course of treatment was classified by type (tertiary, general hospital, and others) and location (capital, metropolitan, or rural) to include the first cancer care quality.^31^ Age was divided by units of 10 years as “≤49 years,” “50–59 years,” “60–69 years,” “70–79 years,” and “≥80 years.”^32^ The types of insurance coverage were divided into three types based on socioeconomic and employment status. Most (approximately 97%) individuals were covered by NHI and were defined as NHI employee or NHI self‐employed. The NHI employee included employees, employers, and their household members, who pay insurance premium based on their salary. The NHI self‐employed includes all other individuals who pay insurance premium based on their income, property, and living standards. The remaining NHI (3%) were defined as medical aid, which includes individuals with a low income or disabilities who do not pay insurance premiums. The insurance premium indicates the socioeconomic level, and is divided into four groups.^4^ In South Korea's cancer care, the cancer‐specific insurance claim code (V193) is designated to medical claim data; NHI beneficiaries pay only 5% for medical costs associated with cancer care, whereas the Medical Aid beneficiaries pay 0%–5% of costs; the others excluding cancer care generally had a co‐payment of 20%–30% under NHI. In this study, CCI was used to consider the clinical severity of the study population. Cancer was excluded from CCI calculation, and medical records related to complications were limited to 365 days from the initial cancer diagnosis date.^33^ The treatment types were defined as the type of cancer treatment—such as surgery, chemotherapy, or radiation therapy—provided to patients within 365 days after diagnosis to adjust the severity of each patient at diagnosis because the claims data did not include cancer staging owing to the nature of the data.^34^, ^35^, ^36^, ^37^

### 2.3 Statistical analysis

To investigate a specific pattern of trajectories for cancer treatment, LCMM was applied to model longitudinally measured cancer care patterns to capture heterogeneity in healthcare utilization of patients with cancer while possibly uncovering group‐specific profiles of response.^38^, ^39^ A specific cancer care pattern of trajectories can be showed as a latent class in a finite mixture where membership in latent classes is modeled with a polychotomous logistic regression. Nonlinear link function was defined to better capture cancer treatment patterns. LCMMs with one to five latent classes were estimated according to the type of cancer. To investigate the cancer care patterns and its outcomes based on the types of cancer, descriptive statistics—including frequencies and percentages or mean and standard deviation—were examined according to the outcome variables. First, the averages of LOS and medical costs were compared, and an analysis of variance was conducted. Second, the frequencies of 5‐year mortality based on the independent variables were compared and chi‐square tests were performed, and a log‐rank test with Kaplan–Meier curves was performed to compare the cumulative mortality by the cancer care patterns. Third, multiple regression analysis was performed using a generalized estimate equation (GEE) model for LOS and medical cost or survival analysis using Cox proportional hazard model for 5‐year mortality, adjusting other covariates to investigate the association between cancer care patterns and its outcomes based on the types of cancer. All statistical analyses in this study were performed using SAS statistical software version 9.4.

## 3 RESULTS

**FIGURE 2:**
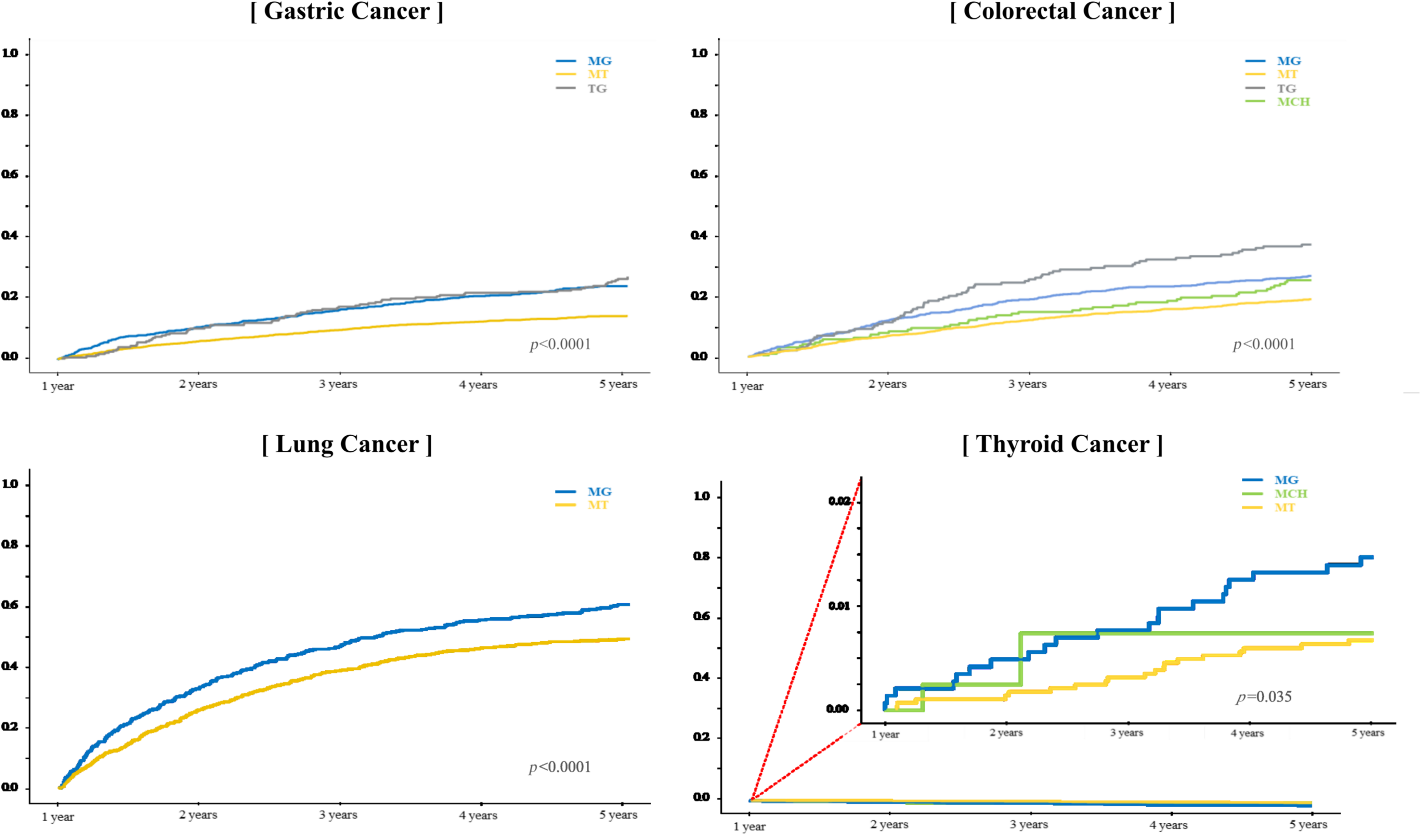
Cumulative mortality by the cancer care patterns according to the type of cancer. † The results of Kaplan–Meier curves and log‐rank test to compare the cumulative mortality by the cancer care patterns. The results of log‐rank test were statistically significant (*p* < 0.0001), excluding those of thyroid cancer. MCH: mainly visiting clinic or hospital, MG: mainly visiting general hospital, MT: mainly visiting tertiary hospital, TG: tertiary to general hospital.

A total of 12,777 patients with cancer were included in this study (gastric: 3353, colorectal: 2915, lung: 1351, and thyroid: 5158). Table 1 shows the results of frequency and percentages of the study population and compares the outcomes including medical cost, LOS, and 5‐year mortality based on the type of cancer. The patterns of gastric cancer were divided into three types (MG, MT, and TG), and those of patients with colorectal cancer were divided into four (MCH, MG, MT, and TG). The results of lung cancer patterns showed two types of cancer care (MG or MT). Thyroid cancer patterns showed three types of cancer care (MCH, MG, or MT). The cancer care patterns demonstrated differences in medical cost and LOS based on cancer type. Patients with MT had the lowest medical cost and LOS than other pattern types in all types of cancer (Tables S2 and S3). With regard to mortality, patients with cancer who mostly visited tertiary hospitals had a lower frequency of 5‐year mortality than other types for all types of cancer (Table S4). Figure 2 shows the results of cumulative mortality according to the cancer care patterns. Patients with MT had lower mortality than other types.

**TABLE 1:** Association between cancer care patterns and outcomes including medical cost, LOS, and 5‐year mortality based on the type of cancer.

Type of Cancer	Patterns of cancer care	Total	Medical cost ($)^a^			LOS (days)^a^			5 years mortality^b^		
			Mean	SD	*p*‐value	Mean	SD	*p*‐value	N	%	*p*‐value
Gastric cancer	MCH	‐	‐	‐	0.1353	‐	‐	0.0034	‐	‐	<0.0001
	MG	1004	4348.1	7381.3		52.1	90.7		241	24.0	
	TG	264	4164.6	6021.0		49.3	75.4		72	27.3	
	MT	2085	3295.1	5494.1		33.8	68.4		298	14.3	
Colorectal cancer	MCH	189	5624.0	6867.0	0.0008	78.2	102.1	<0.0001	47	24.9	<0.0001
	MG	876	5605.7	6766.7		59.3	79.8		234	26.7	
	TG	183	7355.6	8736.9		79.6	99.8		68	37.2	
	MT	1667	5115.3	6116.7		47.4	68.5		316	19.0	
Lung cancer	MCH	‐	‐	‐	0.0178	‐	‐	<0.0001	‐	‐	<0.0001
	MG	438	11,549.6	10,625.9		153.8	144.0		270	61.6	
	TG	‐	‐	‐		‐	‐		‐	‐	
	MT	913	9916.9	10,153.9		107.9	124.8		459	50.3	
Thyroid cancer	MCH	448	962.2	1868.7	0.4699	15.5	29.8	<0.0001	3	0.7	0.0351
	MG	1585	1024.7	1934.6		15.0	28.4		21	1.3	
	TG	‐	‐	‐		‐	‐		‐	‐	‐
	MT	3125	906.7	1038.0		11.5	15.6		19	0.6	‐

^a^ The analysis of variance results comparing the mean and standard deviation of medical cost or LOS based on cancer care patterns.

^b^ The results of chi‐square tests comparing the frequency and percentage of five‐year mortality based on cancer care patterns.

Abbreviations: LOS: length of stay, MCH: mainly visiting clinic or hospital, MG: mainly visiting general hospital, MT: mainly visiting tertiary hospital, SD: standard deviation, TG: tertiary to general hospital.

Table 2 shows the results of the multiple linear regression analysis using GEE model for medical cost or LOS, and the results of survival analysis with Cox proportional hazard models for 5‐year mortality, adjusting covariates to identify the association with cancer care patterns. First, patients with colorectal and lung cancer had significant associations with medical cost based on the patterns. The MCH pattern showed a higher risk of medical costs among patients with colorectal cancer (MCH = RR: 1.143, 95% CI: 1.302–1.599, *p* < 0.0001, MG = RR: 0.988, 95% CI: 0.918–1.063, *p*: 0.7423, TG = RR: 1.040, 95% CI: 0.955–1.133, *p*: 0.3656; ref = MT). Second, with regard to lung cancer, the patterns of MG had 18% higher medical cost than MT. Patients with other cancer care patterns generally had higher LOS than patients with MT patterns. These associations were statistically significant and were observed in the results of patients with all types of cancer in this study, especially in MCH patterns (other than MT, the patterns had 18.6%–155.4% higher LOS values according to cancer types). Patients with gastric cancer with MG or TG patterns had higher mortality than those with the MT pattern (MG = HR: 1.798, 95% CI: 1.319–2.450, *p* = 0.0002, TG = HR: 1.738, 95% CI: 1.333–2.265, *p* < 0.0001; ref = MT). Patients with colorectal cancer who moved to general hospitals from tertiary hospitals had a higher risk of 5‐year mortality than those with the MT pattern (MCH = HR: 1.721, 95% CI: 1.139–2.599, *p*: 0.0099, MG = HR: 1.538, 95% CI: 1.151–2.056, *p*: 0.0036, TG = HR: 1.871, 95% CI: 1.428–2.452, *p* < 0.0001; ref = MT). Patients with lung cancer also showed similar results, with higher risks in MG than MT patterns. However, those with thyroid cancer did not show statistically significant results.

**TABLE 2:** Association between cancer care patterns and outcomes including medical cost, LOS, and five‐year mortality based on the type of cancer.

Outcomes	Patterns of cancer care	Gastric cancer^a^			*p*‐value	Colorectal cancer			*p*‐value	Lung cancer			*p*‐value	Thyroid cancer			*p*‐value
		RR/HR	95% CI			RR/HR	95% CI			RR/HR	95% CI			RR/HR	95% CI		
Medical Cost	MCH	‐	‐	‐	‐	1.443	1.302	1.599	<0.0001	‐	‐	‐	‐	1.018	0.956	1.084	0.5783
	MG	1.003	0.928	1.083	0.9454	0.988	0.918	1.063	0.7423	1.180	1.073	1.297	0.001	1.033	0.987	1.082	0.1616
	TG	1.053	0.974	1.140	0.1957	1.040	0.955	1.133	0.3656	‐	‐	‐	‐	‐	‐	‐	‐
	MT	1.000	‐	‐	‐	1.000	‐	‐	‐	1.000	‐	‐	‐	1.000	‐	‐	‐
LOS	MCH	‐	‐	‐	‐	2.554	2.255	2.892	<0.0001	‐	‐	‐	‐	1.408	1.314	1.510	<0.0001
	MG	1.370	1.260	1.490	<0.0001	1.214	1.109	1.328	<0.0001	1.473	1.316	1.649	<0.0001	1.230	1.169	1.295	<0.0001
	TG	1.386	1.265	1.519	<0.0001	1.186	1.065	1.320	0.0018	‐	‐	‐	‐	‐	‐	‐	‐
	MT	1.000	‐	‐	‐	1.000	‐	‐	‐	1.000	‐	‐	‐	1.000	‐	‐	‐
5 years mortality	MCH	‐	‐	‐	‐	1.721	1.139	2.599	0.0099	‐	‐	‐	‐	0.567	0.130	2.471	0.4503
	MG	1.798	1.319	2.450	0.0002	1.538	1.151	2.056	0.0036	1.400	1.119	1.750	0.003	2.079	0.828	5.219	0.1193
	TG	1.738	1.333	2.265	<0.0001	1.871	1.428	2.452	<0.0001	‐	‐	‐	‐	‐	‐	‐	‐
	MT	1.000	‐	‐	‐	1.000	‐	‐	‐	1.000	‐	‐	‐	1.000	‐	‐	‐

^a^ The multiple linear regression analysis results for medical cost and LOS or survival analysis for 5‐year mortality after controlling for covariates including hospital characteristics (types and location of the hospital that provided the first course of treatment), sex, age, types of insurance coverage, economic status, residence area, Charlson Comorbidity Index, year of diagnosis, and types of cancer treatment.

Abbreviations: HR: hazard ratio; LOS: length of stay, MCH: mainly visiting clinic or hospital, MG: mainly visiting general hospital, MT: mainly visiting tertiary hospital, RR: relative risk, TG: tertiary to general hospital.

## 4 DISCUSSION

The present study examined cancer care patterns after diagnosis of four types of cancer in South Korea and identified cancer care patterns using the LCMM for NHI claims data. These patterns can be defined as MCH, MH, and MT, and each type of cancer showed pattern differences. Gastric and thyroid cancer were classified into three pattern types; however, each pattern had a difference. Additionally, colorectal cancer was classified into four pattern types, whereas lung cancer had two pattern types. In South Korea, most cancer‐related studies commonly focus on the concentration of problems related to medical use of patients with cancer in large hospitals or the metropolitan area. The findings of this study related to patterns of cancer care suggest that the previous classification of cancer care according to whether patients who received cancer care visit large‐sized hospital or hospitals in capital areas cannot capture real‐world situations. Based on this study, future studies should review cancer care patterns related to other factors such as regional distribution.^10^, ^40^, ^41^ Moreover, the classification used in this study may provide a more realistic way of defining the types of cancer care in South Korea, and is expected to be used as a basis for diagnosing cancer treatment problems and preparing alternatives from the healthcare delivery system perspective.

The present study also examined the associations with cancer care patterns and outcomes, including cost, LOS, and mortality after cancer diagnosis. Based on the results of the regression and survival analyses, compared to the patterns of MT, other patterns were associated with higher LOS in all cancer types, and indicated positive associations with higher medical cost in colorectal or lung cancer. In contrast, the survival analysis showed that other types of cancer care have higher mortality than MT patterns. Some healthcare professionals in South Korea argue that the current healthcare delivery system is too skewed toward large hospitals or hospitals in capital areas, resulting in a concentration of patients, thereby causing inequality and inefficiency.^42^, ^43^ However, an analysis of the care patterns and outcomes showed that patients who mainly visited tertiary hospitals received a relatively more efficient cancer care than those who mainly visited smaller medical institutions. Furthermore, this study made various assumptions and has certain limitations; however, it is necessary to highlight issues and alternatives for the cancer care system after reviewing these findings. Under the current structure or system, simply dispersing patients to small hospitals or regional communities is inadequate to be an optimal alternative to resolving the current patient concentration owing to strengthening cancer coverage and concentration in large hospitals in capital areas, it may continue the volume‐outcome relationship in cancer care.^44^, ^45^, ^46^, ^47^ The quantitative growth of infrastructure in cancer control has nearly reached marginal limits through numerous efforts after 2000, and a plan to improve the efficiency of cancer care is necessary. Optimal infrastructure and system to the community or areas that do not have sufficient medical resources have to be developed before the regional distribution of patients with cancer. Owing to limited government resources, “essential medical care” has emerged as a new health policy buzzword in South Korea, but cancer care does not receive the same attention as communicable and other diseases. Rather than being satisfied with the improvement of cancer care, the public's attention is necessary to develop efficient cancer treatments for the burden and impact of cancer. Efforts should be made to develop more effective strategies for cancer control, considering the burden and ripple effect of cancer. Therefore, it is necessary to prepare alternatives after examining patient accessibility and medical quality, and elevated efficiency of cancer care is expected to be achieved by supplying the system with classifications of patients and delivering cancer care based on patients' geographical, socioeconomic, and clinical characteristics.

This study has several strengths and limitations. First, to our knowledge, it is the first to capture cancer care patterns and investigate their association with outcomes in patients with cancer. In this study, based on cancer care after diagnosis, cancer care patterns were defined according to cancer type. Accordingly, the classification of cancer care can be defined, and alternatives can be created based on these patterns. Additionally, this study examined the association between outcomes and cancer care patterns, and the results provided evidence for an alternative health policy perspective. However, due to a lack of detailed information on cancer staging, this study controlled for covariates such as CCI and types of treatment after the diagnosis of cancer to account for disease severity. Therefore, we were unable to examine the potential effects of these factors on the association between cancer care patterns and mortality. Additionally, the nature of the claims data prevented the consideration of important treatment‐related details such as patient motivation and treatment performance. Therefore, it is important to interpret our results with caution and further research is warranted to establish causal relationships. Second, the findings presented in this study cannot be used to establish general associations. Therefore, these results should be interpreted with caution and may not be generalizable to settings beyond South Korea. Third, this was an observational study, not a randomized trial; accordingly, hidden biases could not be fully adjusted. Fourth, the follow‐up period of each patient was intended to be set to 5 years; however, there was a difference in the medical cost and LOS due to early death. Thus, it was converted into a unit of 1 year based on the observation period. Accordingly, there is a possibility of underestimating or overestimating early death patients. Finally, cancer care in South Korea may be affected by supplementary private health insurance owing to the excessive cost burden such as uncovered healthcare services at cancer care. However, such details could not be included in this study owing to the characteristics of the data.^48^

## 5 CONCLUSIONS

Patients who mainly visited large hospitals among the patterns of cancer care had better outcomes even though they did not spend more resources. Considering concerns about the concentration of patients with cancer in large hospitals, the findings of the present study can be used as a basis to address problems and distortions in cancer care in South Korea and improve the efficiency of the medical delivery system over the long term.

## AUTHOR CONTRIBUTIONS

**Dong‐Woo Choi:** Conceptualization (equal); methodology (equal); writing – original draft (equal). **Sun Jung Kim:** Funding acquisition (equal); writing – original draft (equal); writing – review and editing (equal). **SeungJu Kim:** Writing – review and editing (equal). **Dong Wook Kim:** Methodology (equal); writing – review and editing (equal). **Wonjeong Jeong:** Writing – review and editing (equal). **Kyu‐Tae Han:** Conceptualization (equal); formal analysis (equal); funding acquisition (equal); methodology (equal); supervision (equal); writing – original draft (equal).

## FUNDING INFORMATION

This paper was supported by the National Cancer Center (NCC 2210801–2), the Basic Science Research Program through the National Research Foundation of Korea (NRF) funded by the Ministry of Education (2022R1F1A1063423), and by the Soonchunhyang University Research Fund. The funding sources played no role in study design and data interpretation.

## CONFLICT OF INTEREST STATEMENT

The authors declare that they have no conflicts of interest.

## ETHICAL APPROVAL

This study was approved by the Institutional Review Board of National Cancer Center (approval no. NCC2021‐0060). The study was performed in accordance with the Declaration of Helsinki.

## INFORMED CONSENT STATEMENT

This study utilized secondary data, and all patients' personal data were encrypted and anonymized. The approving authority waived the requirement for informed consent due to the use of deidentified patient data.

## CONSENT FOR PUBLICATION

Not applicable.

## Supporting information


Table S1.
Supplementary 2. Supplementary 3. Supplementary 4.

## Data Availability

Data for this study are public data and can be accessed through the following NHIS website (https://nhiss.nhis.or.kr/bd/ab/bdaba000eng.do).
