# Supplementary material for: Cancer care patterns in South Korea: Types of hospital where patients receive care and outcomes using national health insurance claims data
Source: Cancer Med. 2023 May 18;12(13):14707–17. doi: 10.1002/cam4.6093 (PMC10358188; doi:10.1002/cam4.6093)
Supplement: Supplementary file 1 — Table S1. Supplementary 2. Supplementary 3. Supplementary 4. [file CAM4-12-14707-s001.docx]

**Supplementary 1. Classification and distribution of cancer care patterns using trajectory modelling**

| **Type of Cancer** | **Number of patterns** | **Distribution** | | | | | **AIC** | **BIC** |
| --- | --- | --- | --- | --- | --- | --- | --- | --- |
|  |  | **Pattern 1** | **Pattern 2** | **Pattern 3** | **Pattern 4** | **Pattern 5** |  |  |
| Gastric Cancer | 2 | 35.4 | 64.6 |  |  |  | 42,426.1 | 42,476.0 |
|  | **3** | **32.0** | **61.0** | **6.9** |  |  | **40,398.4** | **40,473.3** |
|  | 4 | 4.4 | 28.2 | 6.9 | 60.5 |  | 36,349.3 | 36,449.3 |
|  | 5 | 28.2 | 3.3 | 5.3 | 59.8 | 3.4 | 35,219.0 | 35,344.0 |
| Colorectal Cancer | **2** | **38.8** | **61.2** |  |  |  | **22,754.3** | **22,800.5** |
|  | 3 | 34.9 | 60.9 | 4.3 |  |  | 21,200.1 | 21,269.5 |
|  | 4 | 5.3 | 31.6 | 58.4 | 4.6 |  | 20,551.5 | 20,644.0 |
|  | 5 | 4.1 | 30.8 | 3.6 | 58.0 | 3.5 | 19,574.2 | 19,689.8 |
| Lung Cancer | 2 | 38.2 | 61.8 |  |  |  | 42,331.2 | 42,379.9 |
|  | 3 | 36.5 | 5.3 | 58.2 |  |  | 40,672.2 | 40,745.3 |
|  | **4** | **7.0** | **30.7** | **5.8** | **56.6** |  | **33,523.5** | **33,620.9** |
|  | 5 | 5.8 | 29.0 | 56.1 | 5.1 | 4.0 | 32,165.1 | 32,286.9 |
| Thyroid Cancer | 2 | 37.9 | 62.1 |  |  |  | 73,491.0 | 73,543.4 |
|  | **3** | **8.7** | **30.8** | **60.5** |  |  | **59,603.1** | **59,681.7** |
|  | 4 | 8.6 | 27.6 | 4.6 | 59.3 |  | 55,612.0 | 55,716.9 |
|  | 5 | 7.3 | 27.1 | 3.2 | 58.0 | 4.4 | 53,242.2 | 53,373.3 |

† AIC: Akaike Information Criterion, BIC: Bayesian Information Criterion.

‡ The latent class mixed model results investigated the patterns of hospitals with high medical expenses every three months considering the model fit using BIC and AIC, and only included the model if it consisted of at least 5% of the sample. With these criteria, model fitting and distribution were tested for one to five patterns for each type of cancer.

**Supplementary 2. Medical cost after cancer diagnosis based on types of cancer**

| **Variables** | **Medical cost ($)** | | | | | | | | | | | |
| --- | --- | --- | --- | --- | --- | --- | --- | --- | --- | --- | --- | --- |
|  | **Gastric cancer** | | | **Colorectal cancer** | | | **Lung cancer** | | | **Thyroid cancer** | | |
|  | **Mean** | **SD** | ***P*-value** | **Mean** | **SD** | ***P*-value** | **Mean** | **SD** | ***P*-value** | **Mean** | **SD** | ***P*-value** |
| **Cancer care patterns** |  |  |  |  |  |  |  |  |  |  |  |  |
| MCH | - | - | 0.1353 | 5,624.0 | 6,867.0 | 0.0008 | - | - | 0.0178 | 962.2 | 1,868.7 | 0.4699 |
| MG | 4,348.1 | 7,381.3 |  | 5,605.7 | 6,766.7 |  | 11,549.6 | 10,625.9 |  | 1,024.7 | 1,934.6 |  |
| TG | 4,164.6 | 6,021.0 |  | 7,355.6 | 8,736.9 |  | - | - |  | - | - |  |
| MT | 3,295.1 | 5,494.1 |  | 5,115.3 | 6,116.7 |  | 9,916.9 | 10,153.9 |  | 906.7 | 1,038.0 |  |
| **Sex** |  |  |  |  |  |  |  |  |  |  |  |  |
| Male | 3,702.1 | 6,225.8 | 0.1958 | 5,398.4 | 6,383.6 | 0.1576 | 10,951.3 | 10,514.6 | 0.6067 | 1,081.5 | 2,292.3 | 0.0186 |
| Female | 3,630.6 | 6,077.3 |  | 5,491.9 | 6,852.4 |  | 9,464.8 | 9,910.6 |  | 920.0 | 1,204.5 |  |
| **Age (Years)** |  |  |  |  |  |  |  |  |  |  |  |  |
| ≤49 | 3,763.6 | 6,509.5 | <.0001 | 6,436.1 | 7,432.7 | <.0001 | 11,306.2 | 11,423.9 | <.0001 | 870.5 | 990.2 | 0.5375 |
| 50-59 | 4,173.9 | 6,670.9 |  | 5,825.6 | 7,084.1 |  | 10,881.9 | 10,816.3 |  | 961.6 | 1,765.6 |  |
| 60-69 | 3,292.0 | 5,409.7 |  | 4,955.2 | 5,982.1 |  | 10,907.0 | 10,521.5 |  | 1,062.6 | 1,575.1 |  |
| 70-79 | 3,464.4 | 5,287.6 |  | 5,378.7 | 6,566.3 |  | 9,257.5 | 9,182.7 |  | 1,526.6 | 2,959.5 |  |
| ≥80 | 4,100.6 | 9,722.2 |  | 4,368.5 | 4,868.9 |  | 9,951.7 | 10,238.9 |  | 1,045.0 | 581.8 |  |
| **Residence area** |  |  |  |  |  |  |  |  |  |  |  |  |
| Capital area | 3,863.9 | 6,753.7 | 0.7379 | 5,275.5 | 6,311.1 | 0.3966 | 10,429.2 | 10,995.7 | 0.3826 | 943.1 | 1,589.3 | 0.0722 |
| Metropolitan | 3,577.7 | 5,780.5 |  | 5,449.5 | 6,670.3 |  | 10,725.5 | 10,471.0 |  | 974.5 | 1,611.3 |  |
| Rural | 3,534.0 | 5,731.4 |  | 5,669.4 | 6,880.6 |  | 10,264.4 | 9,289.0 |  | 926.6 | 933.6 |  |
| **Types of insurance coverage** |  |  |  |  |  |  |  |  |  |  |  |  |
| Medical-Aid | 3,387.4 | 4,903.1 | 0.0577 | 5,496.4 | 5,860.8 | 0.0506 | 9,258.6 | 7,425.7 | 0.3138 | 1,102.0 | 1,485.5 | 0.8977 |
| NHI, Self-employed | 3,979.9 | 6,702.1 |  | 5,878.5 | 7,280.7 |  | 11,055.8 | 10,947.9 |  | 997.7 | 1,958.0 |  |
| NHI, Employee | 3,544.7 | 5,964.6 |  | 5,192.4 | 6,199.9 |  | 10,208.4 | 10,137.1 |  | 923.2 | 1,174.6 |  |
| **Economic status** |  |  |  |  |  |  |  |  |  |  |  |  |
| Low | 3,993.4 | 6,865.7 | 0.8883 | 5,296.8 | 5,947.8 | 0.2381 | 10,781.2 | 10,569.3 | 0.5395 | 943.9 | 1,636.3 | 0.1797 |
| Mid-low | 3,720.6 | 6,197.8 |  | 5,646.0 | 6,519.2 |  | 11,303.1 | 11,066.6 |  | 982.6 | 1,272.6 |  |
| Mid-high | 3,716.4 | 5,871.0 |  | 5,509.9 | 7,257.2 |  | 10,161.1 | 9,488.4 |  | 999.1 | 1,824.5 |  |
| High | 3,340.6 | 5,703.3 |  | 5,339.3 | 6,658.8 |  | 9,889.0 | 10,264.5 |  | 892.9 | 1,108.2 |  |
| **Charlson Comorbidity Index (excluding cancer)** |  |  |  |  |  |  |  |  |  |  |  |  |
| ≤1 | 3,154.6 | 5,186.6 | 0.0054 | 4,846.5 | 5,788.0 | 0.0001 | 8,305.8 | 9,057.9 | 0.0002 | 867.5 | 1,005.6 | <.0001 |
| 2 | 3,488.2 | 5,608.9 |  | 5,370.9 | 6,021.4 |  | 11,057.6 | 11,062.8 |  | 940.9 | 1,196.5 |  |
| ≥3 | 4,492.2 | 7,524.4 |  | 6,397.4 | 7,858.0 |  | 11,803.8 | 10,579.1 |  | 1,354.1 | 2,866.1 |  |
| **Year of diagnosis** |  |  |  |  |  |  |  |  |  |  |  |  |
| 2007 | 4,147.4 | 6,193.6 |  | 5,551.0 | 6,577.1 |  | 12,274.8 | 10,226.9 |  | 908.9 | 1,143.5 |  |
| 2008 | 4,159.7 | 6,265.2 |  | 5,858.8 | 6,489.0 |  | 13,430.2 | 12,257.9 |  | 910.3 | 983.0 |  |
| 2009 | 4,687.9 | 6,584.7 |  | 6,261.4 | 6,450.7 |  | 14,433.1 | 10,697.1 |  | 1,068.1 | 1,563.0 |  |
| 2010 | 4,509.1 | 8,289.0 |  | 5,885.6 | 6,512.1 |  | 13,053.4 | 11,669.2 |  | 1,104.5 | 1,503.9 |  |
| 2011 | 4,084.8 | 6,635.8 |  | 5,879.4 | 6,386.6 |  | 11,288.3 | 10,296.7 |  | 1,043.2 | 1,611.2 |  |
| 2012 | 3,128.1 | 5,418.4 |  | 5,224.0 | 7,030.2 |  | 11,155.9 | 9,812.7 |  | 945.5 | 1,895.6 |  |
| 2013 | 3,846.9 | 6,812.9 |  | 5,180.9 | 6,692.0 |  | 9,650.9 | 10,241.7 |  | 955.6 | 1,739.1 |  |
| 2014 | 2,959.3 | 5,232.9 |  | 5,656.9 | 8,048.6 |  | 7,249.0 | 8,966.7 |  | 764.4 | 633.2 |  |
| 2015 | 2,021.1 | 2,755.6 |  | 3,631.2 | 3,545.7 |  | 4,694.9 | 4,573.1 |  | 643.9 | 357.2 |  |
| **Types of cancer treatment** |  |  |  |  |  |  |  |  |  |  |  |  |
| Surgery with chemotherapy or radiation therapy | 7,654.7 | 8,777.6 | <.0001 | 6,906.5 | 6,906.6 | 0.0000 | 11,494.3 | 10,828.0 | <.0001 | 1,147.5 | 1,303.2 | <.0001 |
| Surgery | 1,977.8 | 3,111.5 |  | 2,674.5 | 3,359.5 |  | 3,603.0 | 5,688.0 |  | 661.0 | 710.2 |  |
| Chemotherapy or radiation therapy | 13,654.2 | 10,443.2 |  | 10,590.7 | 10,812.3 |  | 14,311.7 | 10,165.5 |  | 2,457.6 | 5,916.2 |  |
| **Types of first cancer treatment hospital** |  |  |  |  |  |  |  |  |  |  |  |  |
| Tertiary hospital | 4,348.1 | 7,381.3 | 0.2145 | 5,417.3 | 6,617.0 | <.0001 | 10,437.3 | 10,468.9 | 0.0176 | 916.5 | 1,136.9 | 0.9064 |
| General hospital | 3,295.1 | 5,494.1 |  | 5,759.2 | 6,730.9 |  | 10,442.4 | 10,022.1 |  | 1,016.5 | 1,929.4 |  |
| Hospital | 4,164.6 | 6,021.0 |  | 3,756.3 | 4,606.9 |  | 12,139.4 | 8,469.5 |  | 958.4 | 1,849.6 |  |
| **Location of first cancer treatment hospital** |  |  |  |  |  |  |  |  |  |  |  |  |
| Capital area | 3,819.6 | 6,611.8 | 0.2112 | 5,448.1 | 6,610.7 | 0.6283 | 10,018.7 | 10,390.4 | 0.6715 | 944.3 | 1,558.4 | 0.1610 |
| Metropolitan | 3,398.2 | 5,480.9 |  | 5,309.3 | 6,423.4 |  | 11,338.4 | 10,485.0 |  | 926.6 | 1,249.1 |  |
| Rural | 3,603.8 | 5,435.4 |  | 5,611.8 | 6,707.0 |  | 11,223.0 | 9,701.4 |  | 1,014.2 | 1,390.2 |  |
| **Mortality rate** |  |  |  |  |  |  |  |  |  |  |  |  |
| Survived | 1,754.9 | 1,412.5 | <.0001 | 3,158.2 | 2,458.3 | <.0001 | 3,411.6 | 2,800.6 | <.0001 | 873.3 | 772.4 | <.0001 |
| Deceased | 12,313.1 | 10,459.7 |  | 13,144.1 | 9,604.9 |  | 16,448.3 | 10,629.1 |  | 9,807.0 | 10,253.9 |  |
| **Total** | 3,678.9 | 6,177.2 |  | 5,436.3 | 6,576.5 |  | 10,446.2 | 10,333.7 |  | 947.8 | 1,451.8 |  |

† *P*-values for analysis of variance comparing the mean and standard deviation of medical cost based on independent variables.

‡ MCH: Mainly visiting clinic or hospital, MG: Mainly visiting general hospital, TG: Tertiary to general hospital, MT: Mainly visiting tertiary hospital

**Supplementary 3. Length of stay after cancer diagnosis based on the type of cancer**

| **Variables** | **Length of stay (days)** | | | | | | | | | | | |
| --- | --- | --- | --- | --- | --- | --- | --- | --- | --- | --- | --- | --- |
|  | **Gastric cancer** | | | **Colorectal cancer** | | | **Lung cancer** | | | **Thyroid cancer** | | |
|  | **Mean** | **SD** | ***P*-value** | **Mean** | **SD** | ***P*-value** | **Mean** | **SD** | **P-value** | **Mean** | **SD** | ***P*-value** |
| **Cancer care patterns** |  |  |  |  |  |  |  |  |  |  |  |  |
| MCH | - | - | 0.0034 | 78.2 | 102.1 | <.0001 | - | - | <.0001 | 15.5 | 29.8 | <.0001 |
| MG | 52.1 | 90.7 |  | 59.3 | 79.8 |  | 153.8 | 144.0 |  | 15.0 | 28.4 |  |
| TG | 49.3 | 75.4 |  | 79.6 | 99.8 |  | - | - |  | - | - |  |
| MT | 33.8 | 68.4 |  | 47.4 | 68.5 |  | 107.9 | 124.8 |  | 11.5 | 15.6 |  |
| **Sex** |  |  |  |  |  |  |  |  |  |  |  |  |
| Male | 40.3 | 75.1 | 0.0396 | 54.1 | 74.4 | 0.0804 | 127.3 | 132.9 | 0.6184 | 14.5 | 33.0 | 0.0425 |
| Female | 40.9 | 80.1 |  | 56.3 | 81.8 |  | 113.8 | 133.0 |  | 12.6 | 18.7 |  |
| **Age (Years)** |  |  |  |  |  |  |  |  |  |  |  |  |
| ≤49 | 39.8 | 78.9 | <.0001 | 61.8 | 80.9 | <.0001 | 116.4 | 132.1 | <.0001 | 11.4 | 11.2 | 0.0012 |
| 50-59 | 47.0 | 88.4 |  | 60.6 | 86.5 |  | 128.8 | 141.6 |  | 13.8 | 25.1 |  |
| 60-69 | 36.6 | 68.5 |  | 49.6 | 68.8 |  | 126.6 | 132.8 |  | 14.2 | 23.1 |  |
| 70-79 | 38.5 | 69.1 |  | 55.3 | 78.9 |  | 113.4 | 123.9 |  | 24.4 | 62.5 |  |
| ≥80 | 41.6 | 81.6 |  | 43.8 | 63.5 |  | 127.9 | 142.9 |  | 19.0 | 22.2 |  |
| **Residence area** |  |  |  |  |  |  |  |  |  |  |  |  |
| Capital area | 43.0 | 81.3 | 0.8650 | 53.7 | 77.3 | 0.5597 | 124.1 | 136.5 | 0.3069 | 13.4 | 23.9 | 0.0317 |
| Metropolitan | 40.5 | 80.4 |  | 54.2 | 72.6 |  | 122.5 | 130.1 |  | 12.9 | 25.4 |  |
| Rural | 37.4 | 67.5 |  | 57.7 | 82.0 |  | 121.1 | 130.8 |  | 12.3 | 11.4 |  |
| **Types of insurance coverage** |  |  |  |  |  |  |  |  |  |  |  |  |
| Medical-Aid | 45.8 | 79.6 | 0.9433 | 64.9 | 84.3 | 0.1944 | 132.0 | 129.7 | 0.3749 | 16.2 | 27.7 | 0.7636 |
| NHI, Self-employed | 43.3 | 78.1 |  | 60.9 | 86.2 |  | 133.0 | 138.1 |  | 13.5 | 22.4 |  |
| NHI, Employee | 38.8 | 75.9 |  | 51.1 | 71.6 |  | 117.4 | 130.5 |  | 12.6 | 21.4 |  |
| **Economic status** |  |  |  |  |  |  |  |  |  |  |  |  |
| Low | 43.0 | 76.4 | 0.6086 | 54.9 | 73.3 | 0.7340 | 135.1 | 144.5 | 0.4844 | 12.9 | 19.7 | 0.8683 |
| Mid-low | 41.5 | 78.4 |  | 58.6 | 80.7 |  | 135.6 | 137.3 |  | 13.1 | 15.7 |  |
| Mid-high | 40.6 | 75.7 |  | 53.8 | 78.1 |  | 119.6 | 126.0 |  | 13.2 | 27.5 |  |
| High | 37.4 | 76.4 |  | 52.9 | 78.0 |  | 109.1 | 126.6 |  | 12.7 | 22.1 |  |
| **Charlson Comorbidity Index (excluding cancer)** |  |  |  |  |  |  |  |  |  |  |  |  |
| ≤1 | 33.5 | 65.5 | <.0001 | 47.4 | 69.4 | <.0001 | 96.4 | 120.9 | 0.0005 | 11.5 | 16.0 | <.0001 |
| 2 | 36.8 | 67.9 |  | 53.6 | 71.2 |  | 123.4 | 131.1 |  | 12.8 | 13.6 |  |
| ≥3 | 52.3 | 93.3 |  | 67.8 | 90.7 |  | 143.7 | 139.8 |  | 20.2 | 43.3 |  |
| **Year of diagnosis** |  |  |  |  |  |  |  |  |  |  |  |  |
| 2007 | 52.5 | 82.9 | <.0001 | 64.8 | 75.1 | <.0001 | 152.5 | 125.2 | <.0001 | 16.1 | 32.2 | <.0001 |
| 2008 | 51.8 | 87.5 |  | 67.8 | 86.8 |  | 171.6 | 137.6 |  | 13.9 | 13.4 |  |
| 2009 | 56.7 | 92.1 |  | 71.9 | 88.8 |  | 160.8 | 130.0 |  | 15.3 | 25.4 |  |
| 2010 | 49.2 | 82.9 |  | 63.0 | 91.4 |  | 163.6 | 164.2 |  | 15.7 | 30.6 |  |
| 2011 | 42.8 | 74.1 |  | 59.3 | 78.0 |  | 134.8 | 120.1 |  | 14.0 | 16.8 |  |
| 2012 | 33.5 | 67.3 |  | 54.9 | 84.0 |  | 135.0 | 137.2 |  | 12.3 | 20.2 |  |
| 2013 | 43.8 | 86.8 |  | 51.4 | 77.0 |  | 116.9 | 143.5 |  | 12.4 | 23.9 |  |
| 2014 | 27.7 | 64.5 |  | 45.5 | 62.7 |  | 77.2 | 105.4 |  | 8.9 | 11.1 |  |
| 2015 | 14.2 | 28.7 |  | 24.8 | 33.2 |  | 38.0 | 57.7 |  | 6.6 | 3.9 |  |
| **Types of cancer treatment** |  |  |  |  |  |  |  |  |  |  |  |  |
| Surgery with chemotherapy or radiation therapy | 89.0 | 101.1 | <.0001 | 71.1 | 79.0 | <.0001 | 128.4 | 134.0 | <.0001 | 15.8 | 22.4 | <.0001 |
| Surgery | 18.0 | 32.5 |  | 23.0 | 42.5 |  | 29.9 | 46.6 |  | 9.0 | 12.5 |  |
| Chemotherapy or radiation therapy | 188.4 | 150.7 |  | 122.7 | 132.8 |  | 180.3 | 137.1 |  | 32.5 | 71.3 |  |
| **Types of first cancer treatment hospital** |  |  |  |  |  |  |  |  |  |  |  |  |
| Tertiary hospital | 37.5 | 73.3 | 0.1036 | 53.0 | 76.8 | <.0001 | 117.9 | 131.3 | 0.0009 | 12.2 | 19.0 | 0.1626 |
| General hospital | 48.1 | 84.8 |  | 61.7 | 82.0 |  | 133.6 | 136.3 |  | 14.6 | 23.7 |  |
| Hospital | 40.8 | 66.1 |  | 40.8 | 51.2 |  | 230.2 | 141.5 |  | 14.1 | 38.0 |  |
| **Location of first cancer treatment hospital** |  |  |  |  |  |  |  |  |  |  |  |  |
| Capital area | 42.3 | 79.9 | 0.2889 | 54.5 | 78.4 | 0.8228 | 116.9 | 131.1 | 0.6617 | 13.2 | 21.8 | 0.0098 |
| Metropolitan | 37.1 | 72.8 |  | 54.2 | 73.9 |  | 130.8 | 128.9 |  | 12.0 | 19.2 |  |
| Rural | 39.5 | 70.1 |  | 58.8 | 79.6 |  | 140.1 | 147.3 |  | 14.0 | 26.8 |  |
| **Mortality rate** |  |  |  |  |  |  |  |  |  |  |  |  |
| Survived | 17.5 | 26.9 | <.0001 | 29.9 | 36.5 | <.0001 | 34.8 | 48.5 | <.0001 | 12.0 | 14.9 | <.0001 |
| Deceased | 143.7 | 126.8 |  | 140.0 | 111.6 |  | 197.8 | 136.2 |  | 121.0 | 139.2 |  |
| **Total** | 40.5 | 76.8 |  | 55.0 | 77.5 |  | 122.7 | 133.0 |  | 13.0 | 21.8 |  |

† *P*-values for analysis of variance comparing the mean and standard deviation of length of stay based on independent variables.

‡ MCH: Mainly visiting clinic or hospital, MG: Mainly visiting general hospital, TG: Tertiary to general hospital, MT: Mainly visiting tertiary hospital

**Supplementary 4. Distribution of the study population and five-year mortality based on types of cancer**

| **Variables** | **Gastric cancer** | | | | | | **Colorectal cancer** | | | | | | **Lung cancer** | | | | | | **Thyroid cancer** | | | | | |
| --- | --- | --- | --- | --- | --- | --- | --- | --- | --- | --- | --- | --- | --- | --- | --- | --- | --- | --- | --- | --- | --- | --- | --- | --- |
|  | **Total** | **Died** | | **Survived** | | ***P*-value** | **Total** | **Died** | | **Survived** | | ***P*-value** | **Total** | **Died** | | **Survived** | | ***P*-value** | **Total** | **Died** | | **Survived** | | ***P*-value** |
|  |  | ***N*** | **%** | ***N*** | **%** |  |  | ***N*** | **%** | ***N*** | **%** |  |  | ***N*** | **%** | ***N*** | **%** |  |  | ***N*** | **%** | ***N*** | **%** |  |
| **Cancer care patterns** |  |  |  |  |  |  |  |  |  |  |  |  |  |  |  |  |  |  |  |  |  |  |  |  |
| MCH | - | - | - | - | - | <.0001 | 189 | 47 | 24.9 | 142 | 75.1 | <.0001 | - | - | - | - | - | <.0001 | 448 | 3 | 0.7 | 445 | 99.3 | 0.0351 |
| MG | 1,004 | 241 | 24.0 | 763 | 76.0 |  | 876 | 234 | 26.7 | 642 | 73.3 |  | 438 | 270 | 61.6 | 168 | 38.4 |  | 1,585 | 21 | 1.3 | 1,564 | 98.7 |  |
| TG | 264 | 72 | 27.3 | 192 | 72.7 |  | 183 | 68 | 37.2 | 115 | 62.8 |  | - | - | - | - | - |  | - | - | - | - | - |  |
| MT | 2,085 | 298 | 14.3 | 1,787 | 86 |  | 1,667 | 316 | 19.0 | 1,351 | 81.0 |  | 913 | 459 | 50.3 | 454 | 49.7 |  | 3,125 | 19 | 0.6 | 3,106 | 99.4 |  |
| **Sex** |  |  |  |  |  |  |  |  |  |  |  |  |  |  |  |  |  |  |  |  |  |  |  |  |
| Male | 2,265 | 433 | 19.1 | 1,832 | 80.9 | 0.0529 | 1,734 | 393 | 22.7 | 1,341 | 77.3 | 0.8167 | 892 | 510 | 57.2 | 382 | 42.8 | 0.001 | 887 | 13 | 1.5 | 874 | 98.5 | 0.0229 |
| Female | 1,088 | 178 | 16.4 | 910 | 83.6 |  | 1,181 | 272 | 23.0 | 909 | 77.0 |  | 459 | 219 | 47.7 | 240 | 52.3 |  | 4,271 | 30 | 0.7 | 4,241 | 99.3 |  |
| **Age (Years)** |  |  |  |  |  |  |  |  |  |  |  |  |  |  |  |  |  |  |  |  |  |  |  |  |
| ≤49 | 614 | 81 | 13.2 | 533 | 86.8 | <.0001 | 395 | 85 | 21.5 | 310 | 78.5 | <.0001 | 116 | 49 | 42.2 | 67 | 57.8 | <.0001 | 2,761 | 9 | 0.3 | 2,752 | 99.7 | <.0001 |
| 50-59 | 872 | 135 | 15.5 | 737 | 84.5 |  | 693 | 123 | 17.7 | 570 | 82.3 |  | 325 | 155 | 47.7 | 170 | 52.3 |  | 1,501 | 9 | 0.6 | 1,492 | 99.4 |  |
| 60-69 | 1,003 | 161 | 16.1 | 842 | 83.9 |  | 863 | 146 | 16.9 | 717 | 83.1 |  | 483 | 265 | 54.9 | 218 | 45.1 |  | 687 | 12 | 1.7 | 675 | 98.3 |  |
| 70-79 | 723 | 176 | 24.3 | 547 | 75.7 |  | 772 | 228 | 29.5 | 544 | 70.5 |  | 364 | 209 | 57.4 | 155 | 42.6 |  | 194 | 12 | 6.2 | 182 | 93.8 |  |
| ≥80 | 141 | 58 | 41.1 | 83 | 58.9 |  | 192 | 83 | 43.2 | 109 | 56.8 |  | 63 | 51 | 81.0 | 12 | 19.0 |  | 15 | 1 | 6.7 | 14 | 93.3 |  |
| **Residence area** |  |  |  |  |  |  |  |  |  |  |  |  |  |  |  |  |  |  |  |  |  |  |  |  |
| Capital area | 1,355 | 256 | 18.9 | 1,099 | 81.1 | 0.7093 | 1,292 | 284 | 22.0 | 1,008 | 78.0 | 0.4856 | 587 | 305 | 52.0 | 282 | 48.0 | 0.3864 | 2,311 | 16 | 0.7 | 2,295 | 99.3 | 0.5486 |
| Metropolitan | 885 | 157 | 17.7 | 728 | 82.3 |  | 776 | 176 | 22.7 | 600 | 77.3 |  | 323 | 176 | 54.5 | 147 | 45.5 |  | 1,482 | 13 | 0.9 | 1,469 | 99.1 |  |
| Rural | 1,113 | 198 | 17.8 | 915 | 82.2 |  | 847 | 205 | 24.2 | 642 | 75.8 |  | 441 | 248 | 56.2 | 193 | 43.8 |  | 1,365 | 14 | 1.0 | 1,351 | 99.0 |  |
| **Types of insurance coverage** |  |  |  |  |  |  |  |  |  |  |  |  |  |  |  |  |  |  |  |  |  |  |  |  |
| Medical-Aid | 133 | 34 | 25.6 | 99 | 74.4 | 0.0554 | 122 | 45 | 36.9 | 77 | 63.1 | 0.0002 | 40 | 22 | 55.0 | 18 | 45.0 | 0.8111 | 84 | 2 | 2.4 | 82 | 97.6 | 0.1232 |
| NHI, Self-employed | 1,082 | 203 | 18.8 | 879 | 81.2 |  | 982 | 234 | 23.8 | 748 | 76.2 |  | 424 | 234 | 55.2 | 190 | 44.8 |  | 1,501 | 16 | 1.1 | 1,485 | 98.9 |  |
| NHI, Employee | 2,138 | 374 | 17.5 | 1,764 | 82.5 |  | 1,811 | 386 | 21.3 | 1,425 | 78.7 |  | 887 | 473 | 53.3 | 414 | 46.7 |  | 3,573 | 25 | 0.7 | 3,548 | 99.3 |  |
| **Economic status** |  |  |  |  |  |  |  |  |  |  |  |  |  |  |  |  |  |  |  |  |  |  |  |  |
| Low | 874 | 189 | 21.6 | 685 | 78.4 | 0.025 | 767 | 184 | 24.0 | 583 | 76.0 | 0.3023 | 279 | 150 | 53.8 | 129 | 46.2 | 0.2679 | 1,098 | 9 | 0.8 | 1,089 | 99.2 | 0.8177 |
| Mid-low | 793 | 135 | 17.0 | 658 | 83.0 |  | 691 | 162 | 23.4 | 529 | 76.6 |  | 294 | 172 | 58.5 | 122 | 41.5 |  | 1,122 | 10 | 0.9 | 1,112 | 99.1 |  |
| Mid-high | 698 | 122 | 17.5 | 576 | 82.5 |  | 606 | 121 | 20.0 | 485 | 80.0 |  | 324 | 175 | 54.0 | 149 | 46.0 |  | 1,192 | 12 | 1.0 | 1,180 | 99.0 |  |
| High | 988 | 165 | 16.7 | 823 | 83.3 |  | 851 | 198 | 23.3 | 653 | 76.7 |  | 454 | 232 | 51.1 | 222 | 48.9 |  | 1,746 | 12 | 0.7 | 1,734 | 99.3 |  |
| **Charlson Comorbidity Index (excluding cancer)** | |  |  |  |  |  |  |  |  |  |  |  |  |  |  |  |  |  |  |  |  |  |  |  |
| ≤1 | 1,412 | 191 | 13.5 | 1,221 | 86.5 | <.0001 | 1,404 | 263 | 18.7 | 1,141 | 81.3 | <.0001 | 452 | 213 | 47.1 | 239 | 52.9 | 0.001 | 3,538 | 17 | 0.5 | 3,521 | 99.5 | <.0001 |
| 2 | 835 | 141 | 16.9 | 694 | 83.1 |  | 608 | 129 | 21.2 | 479 | 78.8 |  | 339 | 187 | 55.2 | 152 | 44.8 |  | 906 | 9 | 1.0 | 897 | 99.0 |  |
| ≥3 | 1,106 | 279 | 25.2 | 827 | 74.8 |  | 903 | 273 | 30.2 | 630 | 69.8 |  | 560 | 329 | 58.8 | 231 | 41.3 |  | 714 | 17 | 2.4 | 697 | 97.6 |  |
| **Year of diagnosis** |  |  |  |  |  |  |  |  |  |  |  |  |  |  |  |  |  |  |  |  |  |  |  |  |
| 2007 | 314 | 67 | 21.3 | 247 | 78.7 | 0.0122 | 267 | 74 | 27.7 | 193 | 72.3 | 0.3818 | 116 | 74 | 63.8 | 42 | 36.2 | <.0001 | 343 | 3 | 0.9 | 340 | 99.1 | 0.6953 |
| 2008 | 318 | 61 | 19.2 | 257 | 80.8 |  | 279 | 67 | 24.0 | 212 | 76.0 |  | 123 | 74 | 60.2 | 49 | 39.8 |  | 474 | 6 | 1.3 | 468 | 98.7 |  |
| 2009 | 361 | 82 | 22.7 | 279 | 77.3 |  | 314 | 81 | 25.8 | 233 | 74.2 |  | 136 | 90 | 66.2 | 46 | 33.8 |  | 580 | 4 | 0.7 | 576 | 99.3 |  |
| 2010 | 314 | 65 | 20.7 | 249 | 79.3 |  | 306 | 63 | 20.6 | 243 | 79.4 |  | 141 | 81 | 57.4 | 60 | 42.6 |  | 645 | 1 | 0.2 | 644 | 99.8 |  |
| 2011 | 391 | 67 | 17.1 | 324 | 82.9 |  | 327 | 72 | 22.0 | 255 | 78.0 |  | 136 | 79 | 58.1 | 57 | 41.9 |  | 727 | 6 | 0.8 | 721 | 99.2 |  |
| 2012 | 389 | 51 | 13.1 | 338 | 86.9 |  | 342 | 68 | 19.9 | 274 | 80.1 |  | 172 | 91 | 52.9 | 81 | 47.1 |  | 737 | 7 | 0.9 | 730 | 99.1 |  |
| 2013 | 440 | 85 | 19.3 | 355 | 80.7 |  | 342 | 74 | 21.6 | 268 | 78.4 |  | 166 | 83 | 50.0 | 83 | 50.0 |  | 733 | 8 | 1.1 | 725 | 98.9 |  |
| 2014 | 454 | 79 | 17.4 | 375 | 82.6 |  | 396 | 87 | 22.0 | 309 | 78.0 |  | 189 | 83 | 43.9 | 106 | 56.1 |  | 548 | 5 | 0.9 | 543 | 99.1 |  |
| 2015 | 372 | 54 | 14.5 | 318 | 85.5 |  | 342 | 79 | 23.1 | 263 | 76.9 |  | 172 | 74 | 43.0 | 98 | 57.0 |  | 371 | 3 | 0.8 | 368 | 99.2 |  |
| **Types of cancer treatment** |  |  |  |  |  |  |  |  |  |  |  |  |  |  |  |  |  |  |  |  |  |  |  |  |
| Surgery with chemotherapy or radiation therapy | 653 | 256 | 39.2 | 397 | 60.8 | <.0001 | 1,560 | 392 | 25.1 | 1,168 | 74.9 | <.0001 | 356 | 174 | 48.9 | 182 | 51.1 | <.0001 | 2,542 | 17 | 0.7 | 2,525 | 99.3 | <.0001 |
| Surgery | 2,529 | 225 | 8.9 | 2,304 | 91.1 |  | 1,172 | 166 | 14.2 | 1,006 | 85.8 |  | 394 | 68 | 17.3 | 326 | 82.7 |  | 2,481 | 14 | 0.6 | 2,467 | 99.4 |  |
| Chemotherapy or radiation therapy | 171 | 130 | 76.0 | 41 | 24.0 |  | 183 | 107 | 58.5 | 76 | 41.5 |  | 601 | 487 | 81.0 | 114 | 19.0 |  | 135 | 12 | 8.9 | 123 | 91.1 |  |
| **Types of first cancer treatment hospital** | |  |  |  |  |  |  |  |  |  |  |  |  |  |  |  |  |  |  |  |  |  |  |  |
| Tertiary hospital | 2,380 | 389 | 16.3 | 1,991 | 83.7 | <.0001 | 1,886 | 406 | 21.5 | 1,480 | 78.5 | 0.0016 | 972 | 511 | 52.6 | 461 | 47.4 | 0.1152 | 3,398 | 23 | 0.7 | 3,375 | 99.3 | 0.1855 |
| General hospital | 943 | 217 | 23.0 | 726 | 77.0 |  | 881 | 235 | 26.7 | 646 | 73.3 |  | 373 | 213 | 57.1 | 160 | 42.9 |  | 1,509 | 18 | 1.2 | 1,491 | 98.8 |  |
| Hospital | 30 | 5 | 16.7 | 25 | 83.3 |  | 148 | 24 | 16.2 | 124 | 83.8 |  | 6 | 5 | 83.3 | 1 | 16.7 |  | 251 | 2 | 0.8 | 249 | 99.2 |  |
| **Location of first cancer treatment hospital** | |  |  |  |  |  |  |  |  |  |  |  |  |  |  |  |  |  |  |  |  |  |  |  |
| Capital area | 2,004 | 356 | 17.8 | 1,648 | 82.2 | 0.3398 | 1,788 | 388 | 21.7 | 1,400 | 78.3 | 0.1658 | 898 | 453 | 50.4 | 445 | 49.6 | 0.001 | 2,975 | 22 | 0.7 | 2,953 | 99.3 | 0.4511 |
| Metropolitan | 879 | 158 | 18.0 | 721 | 82.0 |  | 724 | 174 | 24.0 | 550 | 76.0 |  | 278 | 166 | 59.7 | 112 | 40.3 |  | 1,536 | 13 | 0.8 | 1,523 | 99.2 |  |
| Rural | 470 | 97 | 20.6 | 373 | 79.4 |  | 403 | 103 | 25.6 | 300 | 74.4 |  | 175 | 110 | 62.9 | 65 | 37.1 |  | 647 | 8 | 1.2 | 639 | 98.8 |  |
| **Total** | **3,353** | **611** | **18.2** | **2,742** | **81.8** |  | **2,915** | **665** | **22.8** | **2,250** | **77.2** |  | **1,351** | **729** | **54.0** | **622** | **46.0** |  | **5,158** | **43** | **0.8** | **5,115** | **99.2** |  |

† *P*-values for chi-square tests comparing the association between independent variables and five-year mortality.

‡ MCH: Mainly visiting clinic or hospital, MG: Mainly visiting general hospital, TG: Tertiary to general hospital, MT: Mainly visiting tertiary hospital.
